# Supplementary material for: Population-scale network embeddings expose educational divides in network structure related to right-wing populist voting
Source: Sci Rep. 2026 Jul 8;16:31398. doi: 10.1038/s41598-026-56690-4 (PMC13645627; doi:10.1038/s41598-026-56690-4)
Supplement: Supplementary file 1 [file 41598_2026_56690_MOESM1_ESM.pdf]

Supplementary Material: Population-Scale Network Embeddings Expose Educational Divides in Network Structure Related to Right-Wing Populist Voting

Datasets and Variables

Registry Data

We used registry datasets provided by Statistics Netherlands. To construct the Dutch population network, we combined the following five datasets:

- **Neighborhood network**. Contains two types of neighborhood relations: (a) persons living in the geographically 10 closest households; (b) persons living in one of 20 households within 200 meters (these were randomly sampled when more than 20 households existed within 200 meters). Documentation: `https://doi.org/10.57934/0b01e410807607b7`
- **Colleagues network**. Contains relations between persons working for the same employer in the same year. When more than 100 people work for the same employer, a subset of the 100 geographically closest colleagues is used. Documentation: `https://doi.org/10.57934/0b01e410807607b1`
- **Family network**. Contains core and extended family relations: Parent, co-parent, grandparent, child, grandchild, full sibling, half sibling, sibling (unknown), full niece/nephew, niece/nephew, uncle/aunt, unmarried partner, married partner, in-law parent, in-law child, in-law sibling, step-parent, step-child, step-sibling, by-marriage full niece/nephew, by-marriage niece/nephew, by-marriage uncle/aunt. Documentation: `https://doi.org/10.57934/0b01e41080760802`
- **Household network**. Contains relations between persons living in the same institutional (e.g., elderly home, student dorm) or non-institutional household (e.g., family home). Documentation: `https://doi.org/10.57934/0b01e4108076077c`
- **Classmate network**. Contains relations between students of the same school, school location, and school year. Relations are indexed by school type: Primary, special, secondary, vocational, higher vocational, university. Documentation: `https://doi.org/10.57934/0b01e4108075f996`

Additionally, we used the following registry datasets to obtain person-level covariates (variable identifiers in parentheses):

- **Basic personal characteristics**. We used this dataset to obtain the variables gender (male, female, unknown; GBAGESLACHT), age (years since birth year; GBAGEBOORTE-JAAR), and number of parents born outside the Netherlands (none, one, both; GBAAAN-TALOUDERSBUITENLAND).
- **Personal income**. From this dataset, we used the variables gross income percentile (INPP100PBRUT), purchasing power (average of income of current and previous year standardized according to prices of current year; INPKKGEM), and change in purchasing power (INPKKMUT).
- **Highest education**. Here, we used highest achieved educational degree (OPLNIV-SOI2016AGG4HBMETNIRWO) based on *Standaard Onderwijsindeling 2021* (`https://www.cbs.nl/nl-nl/onze-diensten/methoden/classificaties/onderwijs-en-beroepen/standaard-onderwijsindeling--soi--/standaard-onderwijsindeling-2021`). We only use the first two numbers of the category codes which results in aggregation level 2 with 5 categories.

Survey Data

We used the Personality and Politics and Values modules of the LISS panel data (wave 16, 2023-2024). From the Personality module (documentation: `https://doi.org/10.57990/c63p-1250`), we selected the following variables (variable identifiers in parentheses):

- Happiness: measured on a scale from 0 to 10 via European Social Survey (ESS) item ”On the whole, how happy would you say you are?” (cp24p010)
- Mood (state): measured on a scale from 1 to 7 via item “How do you feel at the moment?” (cp24p012)
- Mood (trait): measured on a scale from 1 to 7 via item “In general, how do you feel?” (cp24p013)
- Satisfaction with life: measured on a scale from 0 to 10 with the Satisfaction with Life Scale (Diener et al., 1985; cp24p014–018)
- Trust: measured on a scale from 0 to 10 via ESS item “Generally speaking, would you say that most people can be trusted, or that you can’t be too careful in dealing with people?” (cp24p019)
- Extraversion, Neuroticism, Openness, Conscientiousness, Agreeableness (Big 5): measured as sum scores of 10 questions on a scale from 1 to 5 via International Personality Item Pool (`https://ipip.ori.org/`; cp24p020–69)
- Self-esteem: measured as a sum score of 10 questions on a scale from 1 to 7 via Rosenberg Scale (Rosenberg, 2015; cp24p070–79)
- Closeness: measured on a scale from 1 to 7 via Inclusion of Others in the Self scale (Aron et al., 1992; cp24p135)

- Social desirability: measured as a sum score from 10 true/false answers via Marlowe-Crowne Scale (Crowne & Marlow, 1960; cp24p136-145)
- Optimism: measured as a sum score of 10 questions on a scale form 1 to 5 via LOT-R scale (Scheier et al., 1994; cp24p198–207)

From the Politics and Values module (documentation: `https://doi.org/10.57990/xhw0-9614`), we selected the questions:

- "Did you vote in the most recent parliamentary elections, held on 22 November 2023?" (cv24p053)
- "For which party did you vote in the parliamentary elections of 22 November 2023?" (cv24p307)
- "Can you indicate, on a scale from 0 to 10, how much confidence you personally have in each of the following institutions?" with the item "The Dutch government" (cv24p013).

Descriptive Statistics for Linked Dataset

Descriptive statistics of variables in the linked data set (LISS panel survey linked with registry data) are shown in Table 1 and Table 2. Correlations between variables are displayed in Fig. 1.

Table 1: Summary Statistics for Numeric Variables in Linked Dataset

Variable	Mean	SD	Missing
Satisfaction 1	4.97	1.26	1226
Happiness	7.32	1.41	1330
Agreeableness	6.73	4.5	1250
Purch power (log)	10.47	0.41	187
Social desirability	1.13	1.97	1274
Satisfaction 2	5.12	1.27	1226
Satisfaction 5	4.52	1.62	1226
Mood trait	5.57	1.01	1226
Emotional stability	-0.17	3.28	1250
Change purch. power	259372.52	4262177.66	187
Satisfaction 4	5.25	1.27	1226
Extraversion	1.69	6.79	1250
Age	51.99	18.82	NA
Interpersonal trust	5.91	2.31	1333
Optimism	2.49	3.72	1276
Satisfaction 3	5.36	1.19	1226
Closeness	4.44	1.56	1271
Trust government	4.73	2.28	709
Openness	4.72	3.48	1250
Conscientiousness	6.02	4.91	1250
Mood state	5.41	1.18	1225
Income percentile	54.92	28.1	284
Self esteem	14.14	10.74	1253

Total sample size was 6063. NA = value not published for privacy reasons.

Table 2: Summary Statistics for Categorical Variables in Linked Dataset

Variable	Level	n	Proportion
Parents born abroad	Zero	NA	NA
	Both	816	0.1346
	One	529	0.0873
	Missing	NA	NA
Gender	Female	3254	0.5367
	Male	NA	NA
	Missing	NA	NA
General voting	GL/PvdA	941	0.1552
	PVV	855	0.141
	Missing	826	0.1362
	Vvd	683	0.1127
	NSC	628	0.1036
	Not voted	520	0.0858
	D66	310	0.0511
	CDA	200	0.033
	SP	186	0.0307
	BBB	155	0.0256
	PvdD	152	0.0251
	Not elligible	136	0.0224
	CU	103	0.017
	Volt	87	0.0143
	SGP	78	0.0129
	Other	64	0.0106
	DENK	49	0.0081
	FvD	47	0.0078
	JA21	27	0.0045
	Blank	16	0.0026
Voted	Yes	4685	0.7727
	Missing	722	0.1191
	No	520	0.0858
	Not eligible	136	0.0224
Right-wing populist voting	Non populist	3497	0.5768
	Populist	1084	0.1788
	Missing	826	0.1362
	Not voted	520	0.0858
	Not eligible	136	0.0224
Highest achieved education	Missing	1877	0.3096
	Higher vocational	1564	0.258
	Bachelor	1200	0.1979
	Master/doctorate	762	0.1257
	Vocational	470	0.0775
	Primary	190	0.0313

Total sample size was 6063. NA = value not published for privacy reasons.

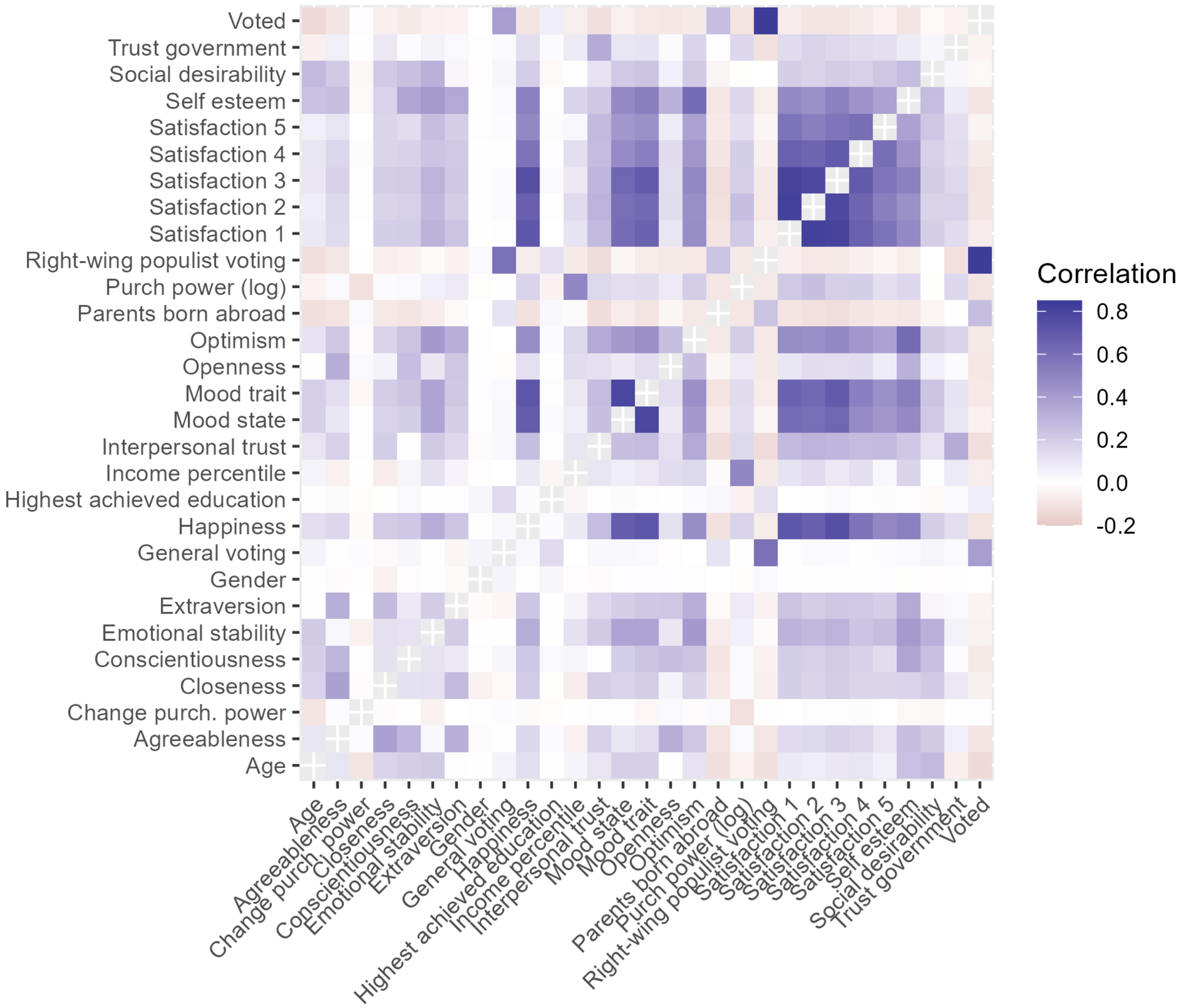

Figure 1: **Correlations between variables in the linked dataset**. The total sample size was 6063. For pairs of numerical variables, Pearson correlations were calculated. For pairs of numerical and categorical variables, point-biserial correlations were calculated. For pairs of categorical variables, Cramer's V was calculated.

Prediction Algorithm Hyperparameter Ranges

Table 3: Prediction Algorithm Hyperparameter Ranges

Algorithm	Hyperparameter	Lower	Upper
Logistic regression	L2 regularization (inverse)[a]	0.001	100
K-nearest neighbors	Number of neighbors	2	100
XGBoost	Learning rate	0	1
	Maximum depth	2	50
	Minimum child weight	1	100
	Subsample rows	0.2	1
	Subsample columns (by tree)	0.2	1
	Subsample columns (by level)	0.2	1
	L2 regularization[a]	0.001	100

[a] Values were sampled on the log scale.

Posterior Predictive Checks

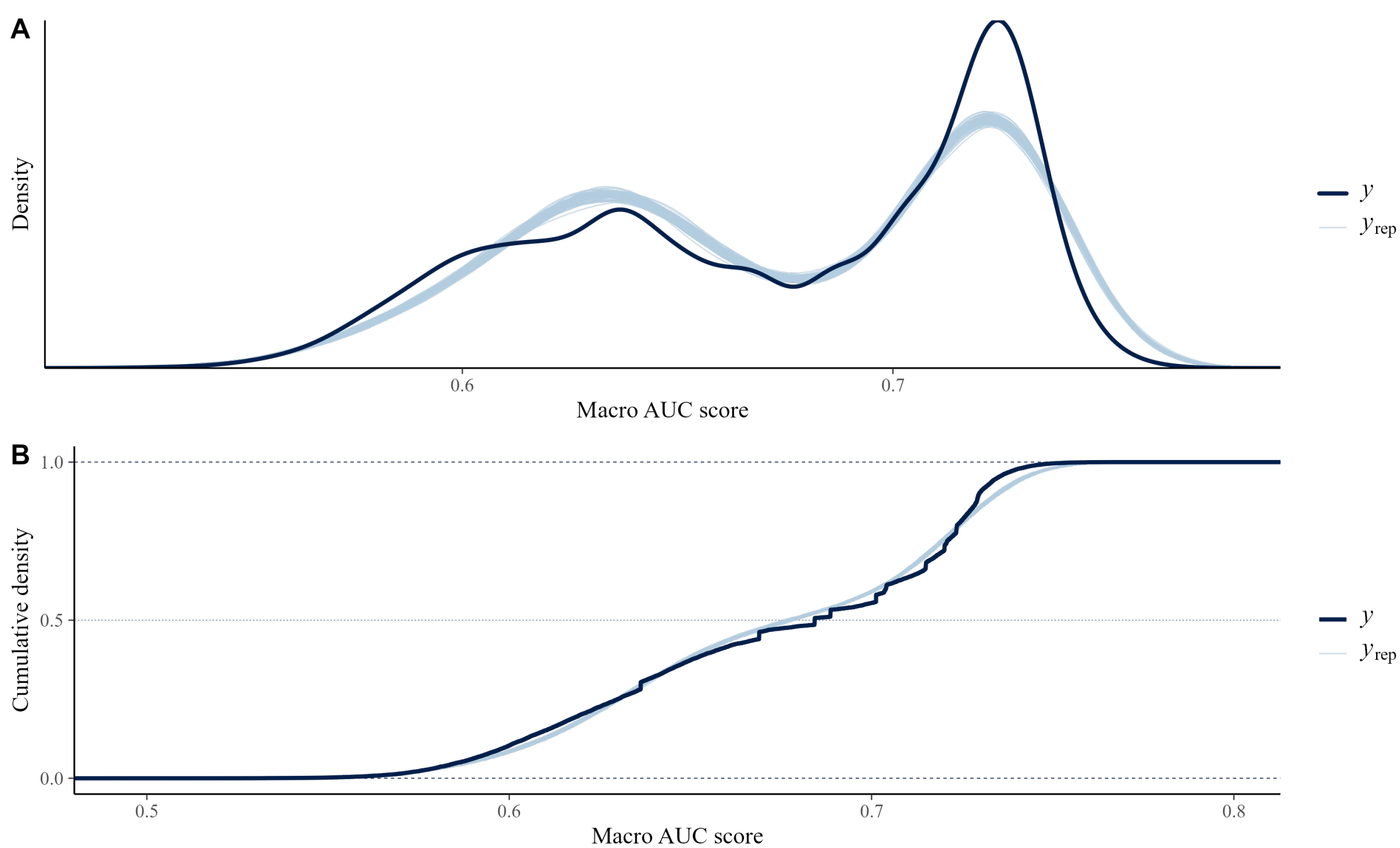

Figure 2: **Posterior predicted and observed macro AUC prediction performance scores for right-wing populist voting**. y indicates observed and y_{rep} the predicted scores.

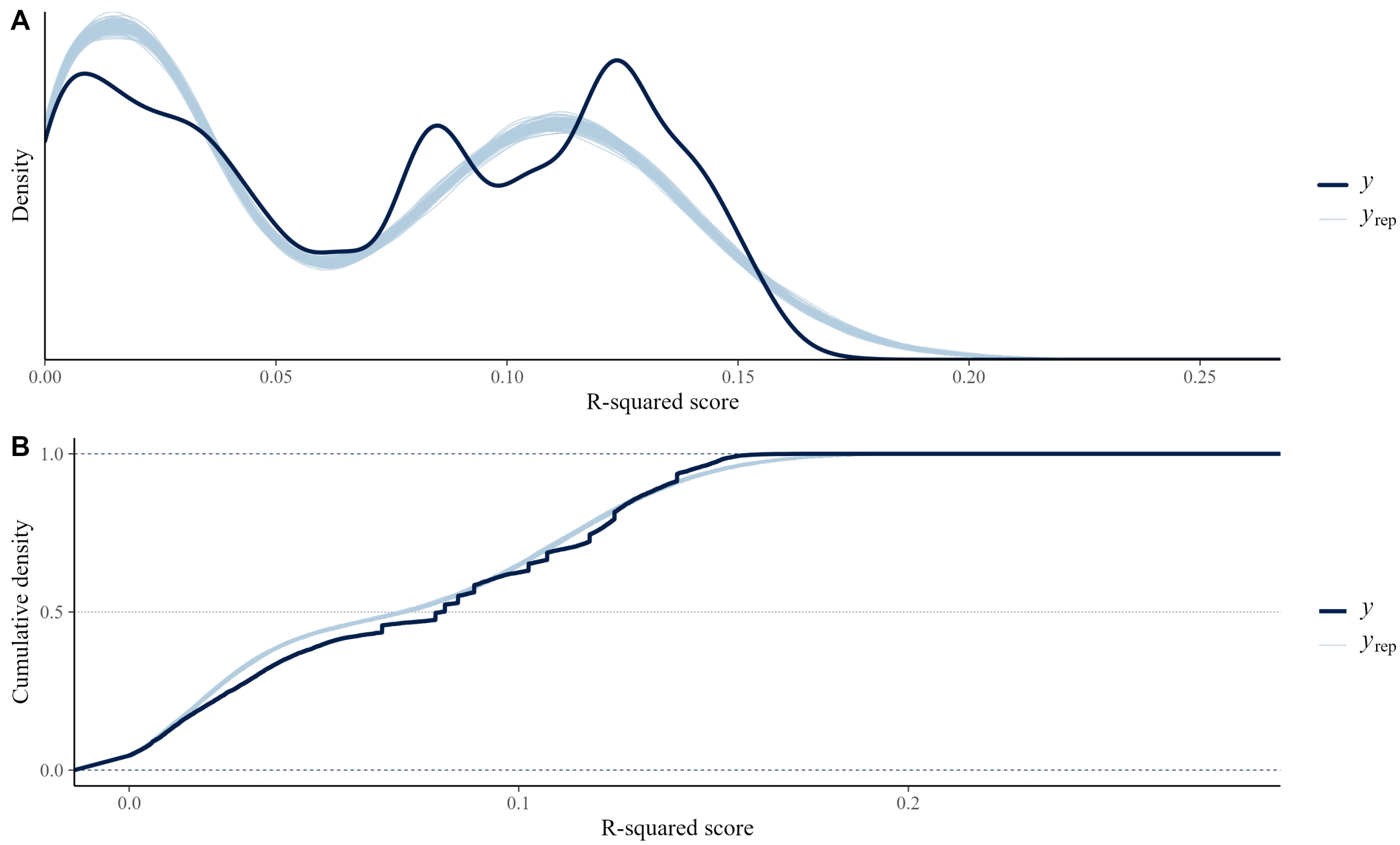

Figure 3: **Posterior predicted and observed R-squared prediction performance scores for trust in the government**. y indicates observed and y_{rep} the predicted scores.

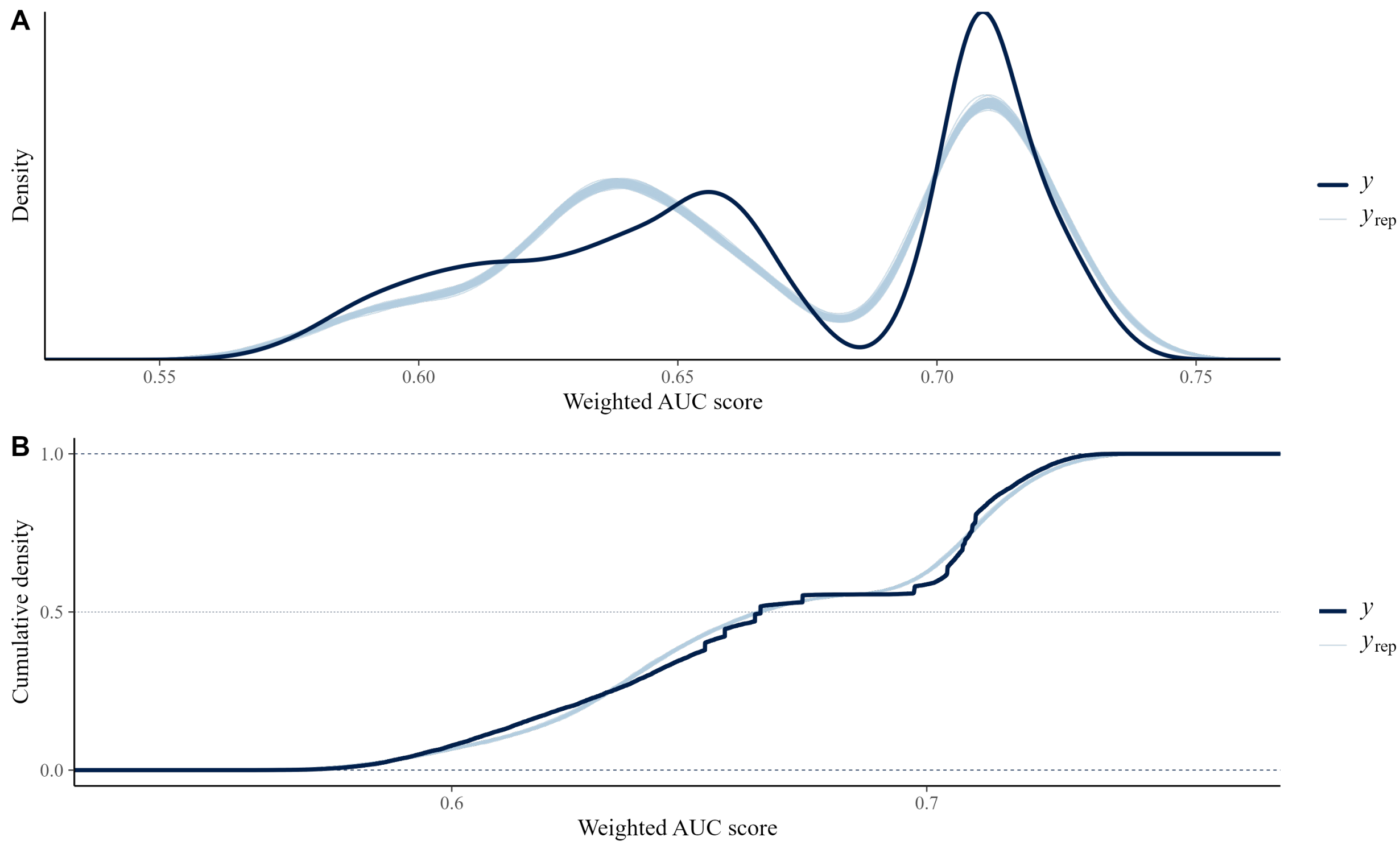

Figure 4: **Posterior predicted and observed weighted AUC prediction performance scores for general voting**. y indicates observed and y_{rep} the predicted scores.

We assessed the fit of the Bayesian regression models by comparing their posterior predictive distributions to the observed prediction scores. For right-wing populist voting, the comparison is shown in Fig. 2, for trust in the government in Fig. 3, and for general voting in Fig. 4. For all three outcomes, the regression model captured the distribution of the observed prediction scores well with some minor deviations.

Prediction Performance

Right-Wing Populist Voting

Marginal effects of all manipulations on estimated out-of-sample prediction scores for right-wing populist voting are shown in Table 4.

Table 4: Simple Contrasts for Prediction Peformance Scores

Contrast			95% CI	
Level 1	Level 2	Difference	Lower	Upper
Estimator				
LinReg	KNN	**0.047**	0.046	0.047
XGB	KNN	**0.058**	0.058	0.059
XGB	LinReg	**0.012**	0.011	0.012
Feature set				
Embed	Cov	**-0.09**	-0.09	-0.089
Embed + Cov	Cov	**-0.012**	-0.013	-0.012
Embed + Cov	Embed	**0.077**	0.077	0.078
Year				
2021	2020	0.00014	-0.00069	0.00096
2022	2020	-0.00026	-0.0011	0.00059
2022	2021	-0.0004	-0.0012	0.00043
DINE-transformed				
Yes	No	-0.0005	-0.0012	0.00016
DeepWalk walk length				
20	10	**0.0018**	0.00089	0.0027
5	10	**-0.0091**	-0.01	-0.0083
5	20	**-0.011**	-0.012	-0.01
DeepWalk walks per node				
100	10	**0.0073**	0.0065	0.008
DeepWalk window size				
5	2	0.00042	-0.00043	0.0013
8	2	-0.00039	-0.0013	0.00046
8	5	-0.00081	-0.0017	0.000054
Method				
DeepWalk	LINE	**0.006**	0.0045	0.0076
Dimension				
24	16	0.0032	-0.00034	0.0067
8	16	**-0.01**	-0.014	-0.0068
8	24	**-0.014**	-0.017	-0.01
32	128	**0.0077**	0.0067	0.0086
64	128	**0.0036**	0.0027	0.0045
64	32	**-0.004**	-0.0048	-0.0032

Differences in macro AUC score. Positive difference values indicate that the marginal predictions for level 1 are higher than those for level 2. Differences for which the 95% quantile credible interval (CI) excludes zero are highlighted in bold. KNN = k-nearest neighbor. LinReg = linear regression. XGB = XGBoost. Embed = embeddings. Cov = covariates.

Trust in the Government

For the outcome trust in the government, we removed 709 participants with missing responses in the LISS Panel data (11.7%). In the covariates feature set, we included the same variables as for right-wing populist voting (only trust in the government was excluded because it is the outcome here). We also included whether participants voted (yes, no, missing) as a predictor. To assess out-of-sample prediction performance, we computed the R^2 score between predicted and observed

trust in government responses. Because some R^2 scores were below zero, we truncated them to zero, and used a zero-inflated beta family in the Bayesian regression to predict them. We removed missing scores because our implementation did not allow missing values when using the zero-inflated beta family.

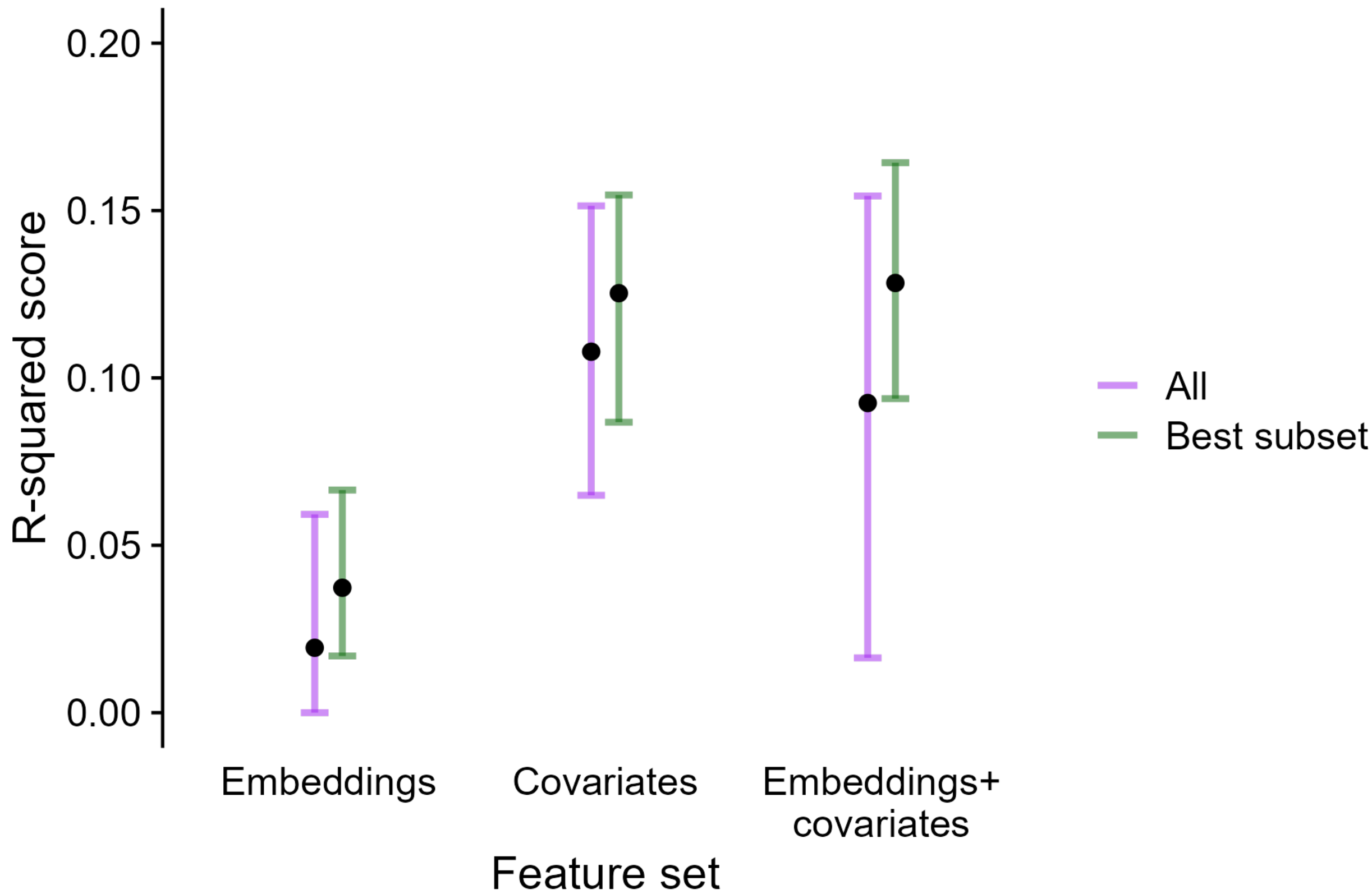

Figure 5: **Out-of-sample prediction performance for trust in the government**. Performance was measured with the R-squared score (on y-axis). The x-axis shows different feature sets used for prediction. Points indicate the posterior predictive mean. Vertical bars indicate 95% credible intervals. Colors indicate whether the predictions were made for all scores (purple) or only for a subset of the best performing prediction models (XGBoost and embeddings with 100 walks per node of length 20 and 32 embedding dimensions, green).

We compared the out-of-sample prediction scores for trust in the government between features sets (embeddings-only, covariates-only, embeddings-plus-covariates) in Fig. 5. We observed that aggregated over all prediction models, embeddings-only models performed above chance-level ($R^2 > 0$), but worse than covariates-only, $\Delta R^2 = 0.085$, 95% CI $[0.084, 0.086]$, and embeddings-plus-covariates models, $\Delta R^2 = 0.07$, 95% CI $[0.069, 0.070]$. Covariates-only models performed slightly better than embeddings-plus-covariates models, $\Delta R^2 = -0.015$, 95% CI $[-0.016, -0.015]$. When selecting the best subset of prediction models, embeddings-plus-covariates models performed slightly better than covariates-only models, $\Delta R^2 = 0.017$, 95% CI $[0.015, 0.020]$. The marginal effects of all other manipulations are shown in Table 5.

Table 5: Contrasts for Trust in the Government Prediction Peformance Scores

Contrast			95% CI	
Level 1	Level 2	Difference	Lower	Upper
Estimator				
LinReg	KNN	**0.037**	0.037	0.038
XGB	KNN	**0.046**	0.045	0.047
XGB	LinReg	**0.0086**	0.0077	0.0093
Feature set				
Embed	Cov	**-0.085**	-0.086	-0.084
Embed + Cov	Cov	**-0.015**	-0.016	-0.015
Embed + Cov	Embed	**0.07**	0.069	0.07
Year				
2021	2020	**0.002**	0.0012	0.0027
2022	2020	**0.0011**	0.00034	0.0019
2022	2021	**-0.00089**	-0.0017	-0.00012
DINE-transformed				
Yes	No	-0.00005	-0.0007	0.00061
DeepWalk walk length				
20	10	**0.003**	0.0022	0.0039
5	10	**-0.0059**	-0.0067	-0.0051
5	20	**-0.0089**	-0.0097	-0.008
DeepWalk walks per node				
100	10	**0.0023**	0.0017	0.003
DeepWalk window size				
5	2	**0.0011**	0.00028	0.0018
8	2	0.00036	-0.00045	0.0012
8	5	-0.0007	-0.0015	0.00013
Method				
DeepWalk	LINE	0.00019	-0.0012	0.0015
Dimension				
24	16	**0.0012**	-0.0022	0.0047
8	16	**-0.005**	-0.0082	-0.0018
8	24	**-0.0061**	-0.0095	-0.0028
32	128	**0.0086**	0.0078	0.0094
64	128	**0.0063**	0.0055	0.0071
64	32	**-0.0023**	-0.0031	-0.0014

Differences are in R-squared score. Positive difference values indicate that the marginal predictions for level 1 are higher than those for level 2. Differences for which the 95% quantile credible interval (CI) excludes zero are highlighted in bold. KNN = k-nearest neighbor. LinReg = linear regression. XGB = XGBoost. Embed = embeddings. Cov = covariates.

General Voting

For general voting, computed the weighted AUC to assess out-of-sample prediction performance. The feature sets and Bayesian regression model were the same as for right-wing populist voting except that the outcome had 19 levels (17 parties, missing, not-voted).

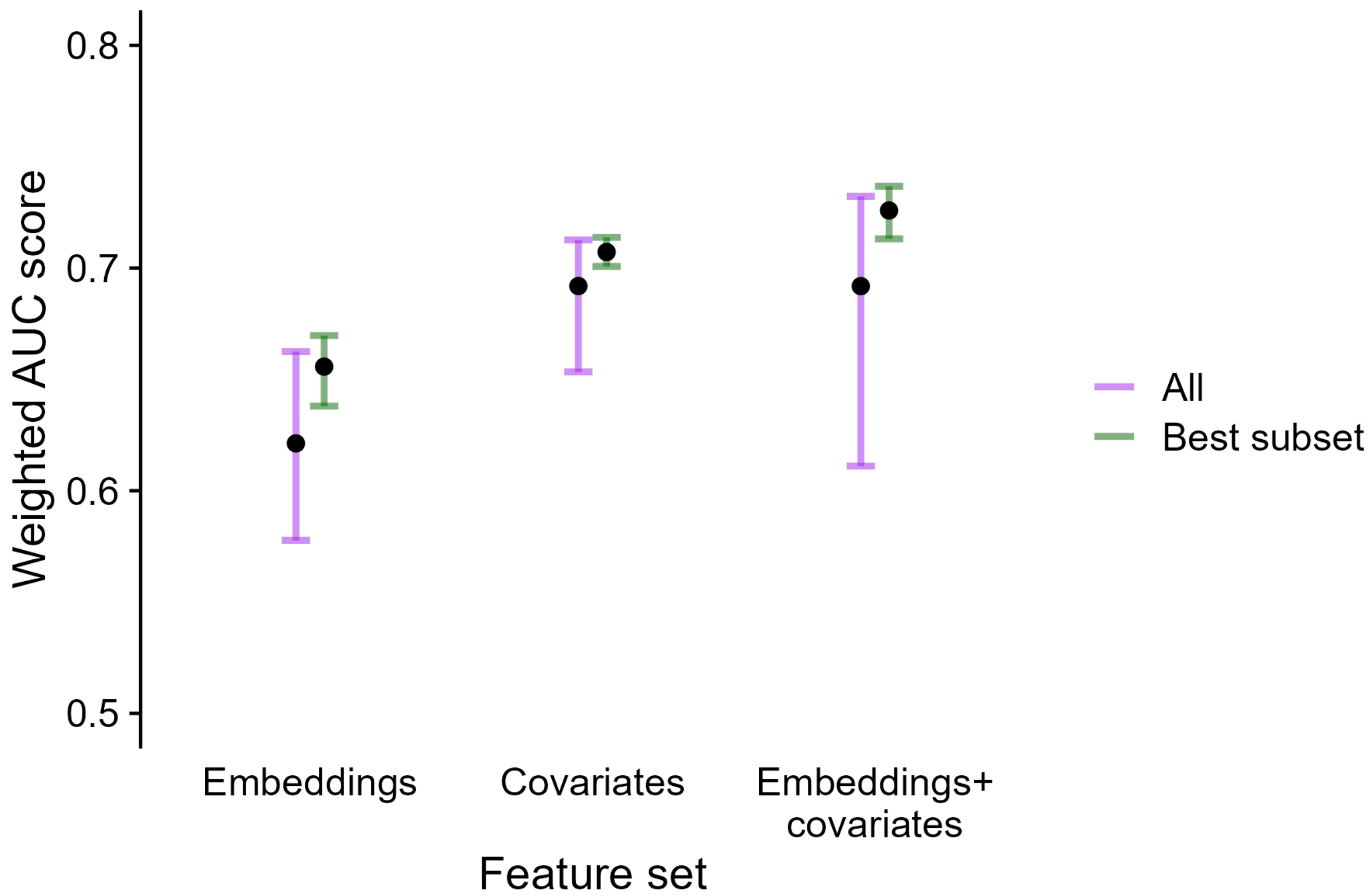

Figure 6: **Out-of-sample prediction performance for general voting**. Performance was measured with the weighted AUC score (on y-axis). The x-axis shows different feature sets used for prediction. Points indicate the posterior predictive mean. Vertical bars indicate 95% credible intervals. Colors indicate whether the predictions were made for all scores (purple) or only for a subset of the best performing prediction models (XGBoost and embeddings with 100 walks per node of length 20 and 32 embedding dimensions, green).

We also compared the out-of-sample prediction scores for general voting between features sets (Fig. 6). When aggregating over all prediction models, embeddings-only models performed above chance-level (AUC > 0.5), but worse than covariates-only, ΔAUC $= 0.071$, 95% CI $[0.070, 0.071]$, and embeddings-plus-covariates models, ΔAUC $= 0.071$, 95% CI $[0.070, 0.071]$. Covariates-only models performed similar embeddings-plus-covariates models, ΔAUC $= 0.00003$, 95% CI $[-0.0004, 0.0004]$. When selecting the best subset of prediction models, embeddings-plus-covariates models performed slightly better than covariates-only models, ΔAUC $= 0.024$, 95% CI $[0.023, 0.024]$. The marginal effects of all other manipulations are shown in Table 6.

Table 6: Contrasts for General Voting Prediction Peformance Scores

Contrast			95% CI	
Level 1	Level 2	Difference	Lower	Upper
Estimator				
LinReg	KNN	**0.051**	0.051	0.051
XGB	KNN	**0.053**	0.053	0.054
XGB	LinReg	**0.0022**	0.0018	0.0025
Feature set				
Embed	Cov	**-0.071**	-0.071	-0.07
Embed + Cov	Cov	0.000034	-0.00037	0.00043
Embed + Cov	Embed	**0.071**	0.07	0.071
Year				
2021	2020	**0.0013**	0.0008	0.0018
2022	2020	0.00042	-0.000093	0.00092
2022	2021	**-0.00085**	-0.0014	-0.00035
DINE-transformed				
Yes	No	**-0.0007**	-0.0011	-0.00029
DeepWalk walk length				
20	10	**0.0052**	0.0047	0.0057
5	10	**-0.0098**	-0.01	-0.0093
5	20	**-0.015**	-0.016	-0.014
DeepWalk walks per node				
100	10	**0.0079**	0.0075	0.0083
DeepWalk window size				
5	2	**0.0016**	0.0011	0.0021
8	2	**0.00054**	0.000014	0.0011
8	5	**-0.0011**	-0.0016	-0.00054
Method				
DeepWalk	LINE	**0.0054**	0.0045	0.0063
Dimension				
24	16	**0.0036**	0.0014	0.0058
8	16	**-0.014**	-0.016	-0.011
8	24	**-0.017**	-0.019	-0.015
32	128	**0.0091**	0.0086	0.0096
64	128	**0.0056**	0.0051	0.0062
64	32	**-0.0035**	-0.004	-0.0029

Differences are in weighted AUC score. Positive difference values indicate that the marginal predictions for level 1 are higher than those for level 2. Differences for which the 95% quantile credible interval (CI) excludes zero are highlighted in bold. KNN = k-nearest neighbor. LinReg = linear regression. XGB = XGBoost. Embed = embeddings. Cov = covariates.

Selected Prediction Model Configurations

Table 7: Configurations of Prediction Models Used in Exploratory and Sensitivity Analyses

	Model exploratory analysis	Model sensitivity analysis
Feature set	Embeddings + covariates	Embeddings + covariates
Algorithm	XGBoost	XGBoost
DINE-transformed	Yes	Yes
Network year	2021	2022
Embedding method	DeepWalk	DeepWalk
Walks per node	100	100
Walk length	20	20
Window size	5	2
Dimensions	32	32
Mean test macro AUC	0.743	0.739

Sensitivity Analysis

Here, we present exploratory results for the second best prediction models using DINE-transformed embeddings (see Table 7 for details). For feature importance scores measured by SHAP values, see Fig. 7. For edge utilities across different network ties, see Fig. 8. For edge utility strength, see Fig. 9. For municipality level edge utilities, see Fig. 10.

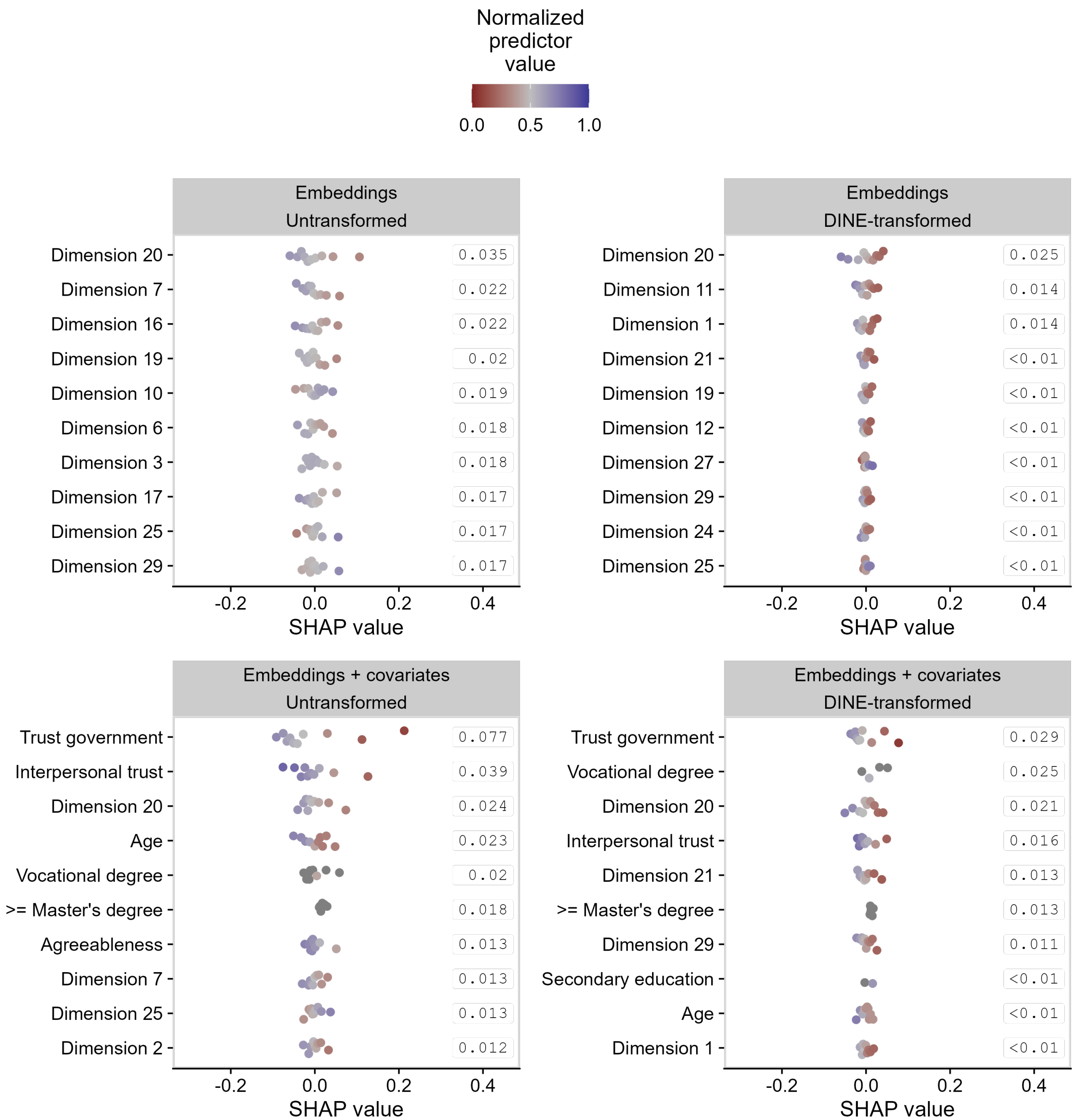

Figure 7: **Importance of 10 most important variables predicting populist voting behavior in the LISS panel data**. Importance was quantified with SHAP values for each predictor and observation. Individual SHAP values were aggregated for each decile to guarantee privacy of the panel subjects. Each point represents the average SHAP value of a decile. Color indicates the average SHAP value in the decile, normalized between zero and one. Grey-colored points indicate that the average value could not be published to prevent group disclosure. Predictors on the y-axis are ordered according to the mean of their absolute decile-averaged SHAP values which are displayed on the right side of each panel. Panels contain results for predictor sets that included (A) only embeddings or (B) embeddings and covariates. The results with untransformed versus DINE-transformed embeddings is shown in the two columns.

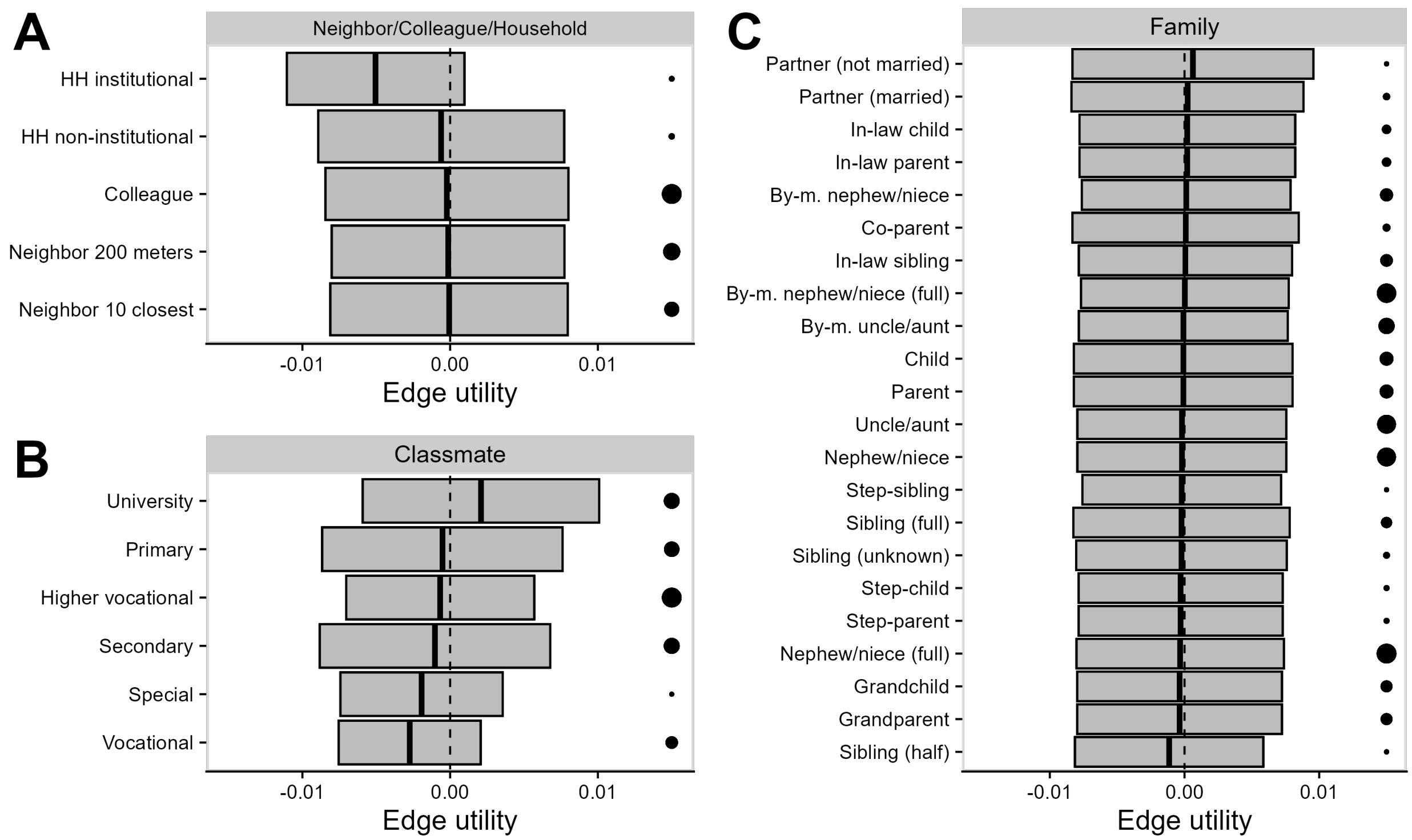

Figure 8: **Edge utility scores (x-axis) for different network relation types (y-axis) and relation groups**. Bold vertical bars indicate means and boxes span ± 2 standard deviations from the mean of the edge utility distributions of (A) neighbors, colleagues and households, (B) classmates, and (C) family relations. The size of the circles on the right of each box indicate the number of relations of the respective type. Classmate relations typically refer to students enrolled at the same school in the same location in the same school year (but this differs per relation type). Special schools are dedicated to students with mental, physical, or learning disabilities. Examples for institutional households are elderly homes, student dorms, and prisons. For neighbor relations, 200 meters refers to 20 random persons living within a 200 meter radius and 10 closest refers to 10 random persons living at the closest addresses. HH = household. By-m. = related by marriage.

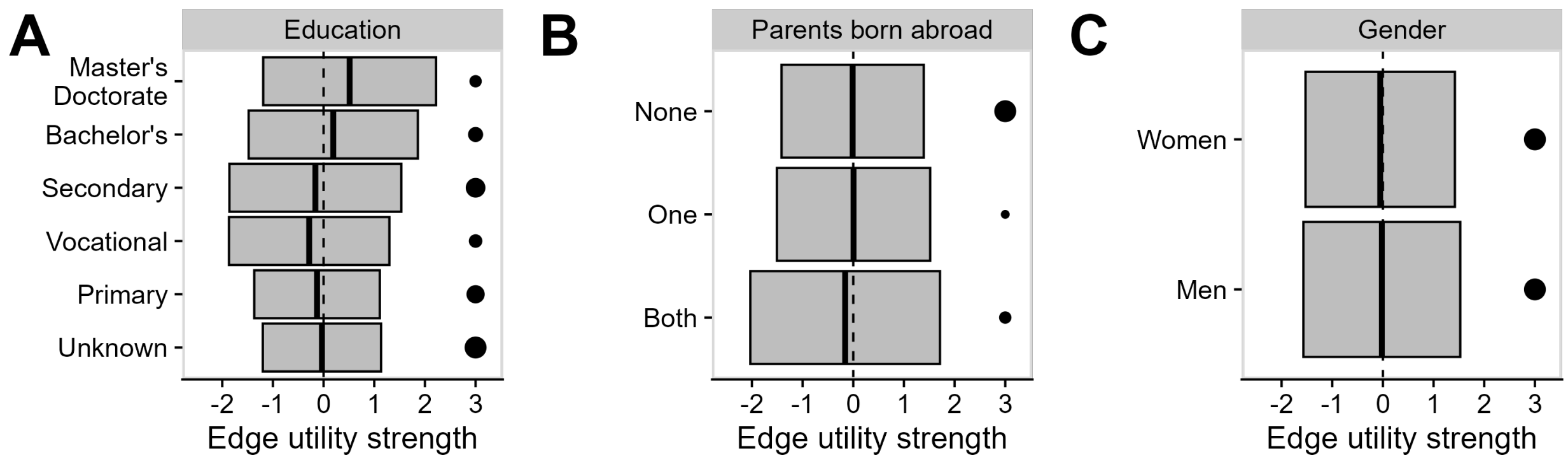

Figure 9: **Edge utility strength for different person-level variables**. Highest achieved education levels (A), the number of parents born outside the Netherlands (B), and gender (C). Bold vertical bars indicate means and boxes span $\pm$ 2 standard deviations from the mean of the edge utility strength distributions. The size of the circles on the right of each box indicate the number of persons in the respective category. The categories are mutually exclusive within each panel but not across panels.

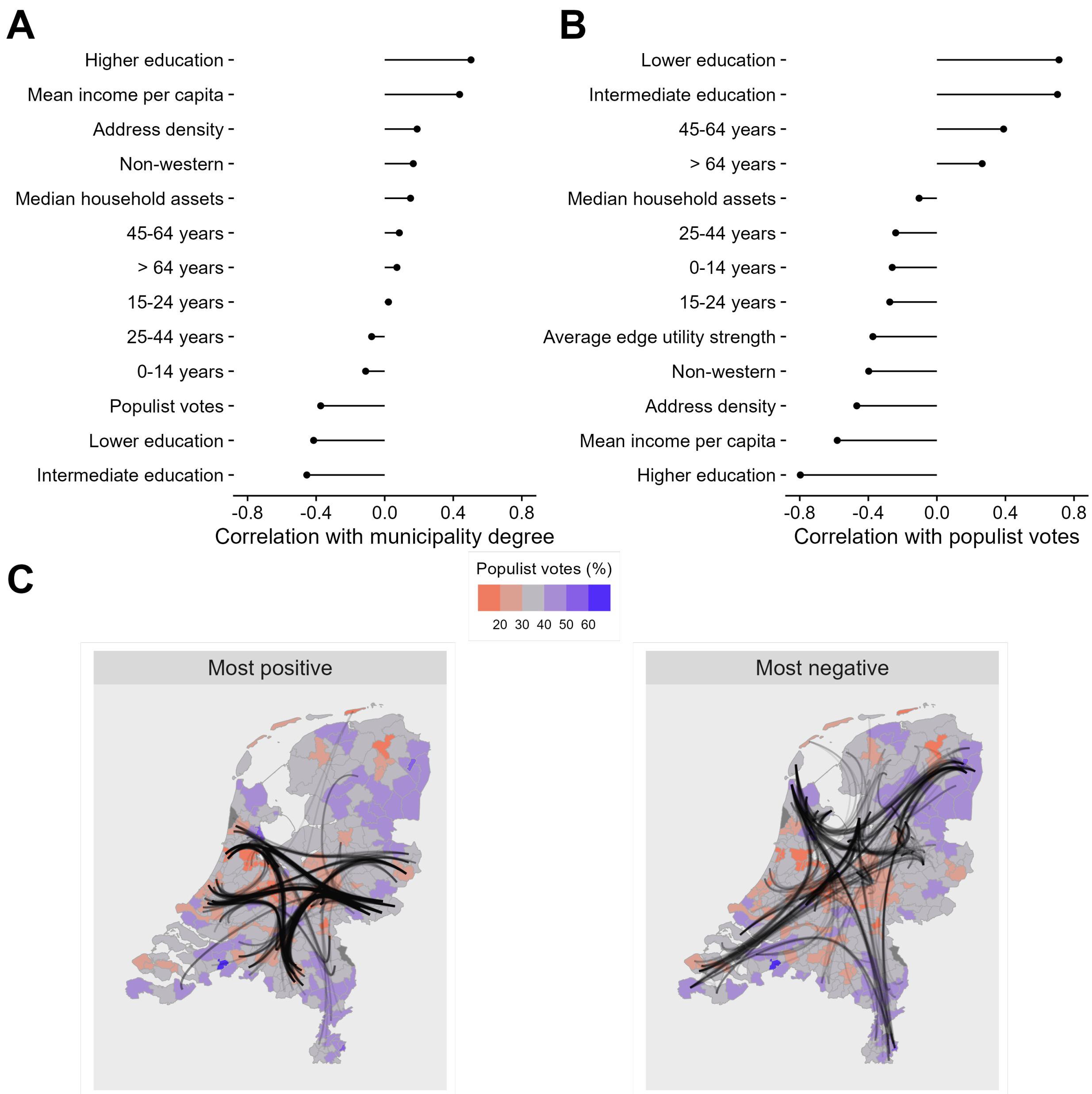

Figure 10: **Average edge utility strength at the municipality level**. Pearson correlations between aggregated municipality statistics and (A) municipality average edge utility strength and (B) right-wing populist votes. C: Maps of the Netherlands and average edge utility connections between Dutch provinces. The right panel shows the most positive connections (top percentile of average edge utility distribution) and the left panel shows the most negative connections (bottom percentile). Color indicates the percentage of votes for a right-wing populist party in each province (gray represents the country-wide average).

Deviations From Preregistration

We assess the reader impact of deviations from our preregistration through changes in the severity of testing our hypotheses and the validity of our inferences (Lakens, 2024). Test severity refers

to controlling the probability of making an incorrect scientific claim, for example, by exploiting researcher degrees of freedom, whereas validity is about the truth-likeness of inferences. Adapted from the preregistration deviations template by Willroth and Atherton (2024).

1. **Type**: Hypotheses
   Reason: Plan not possible
   Timing: After data access
   Original wording: We expect the following local graph substructures to emerge: Densely connected clusters ("hubs") that are connected via "wide" bridges, that is, multiple nodes that connect the clusters.
   Deviation: This hypothesis turned out to be too difficult to test given the size of the population network and our available computational methods and resources.
   Reader impact: The results for which this hypothesis was intended are now reported as exploratory to clearly indicate that the analysis was not pre-registered.

2. **Type**: Analysis
   Reason: New Knowledge
   Timing: After data access
   Original wording: We will train Skip-Gram models until the training loss increases and at most 20 epochs.
   Deviation: We realized that our Skip-Gram implementation (from the gensim Python package) suffers from a floating-point precision problem when tracking the training loss. We also encountered this issue in our dataset, rendering training loss unreliable. Instead, we chose to train for a fixed number of epochs dependent on our compute budget. All Skip-Gram models with 10 walks per node were trained for 20 epochs. All models with 100 walks per node were trained for 2 epochs. This was the maximum we could train for given our computational resources.
   Reader impact: Because we saw only small differences in the predictive performance of embeddings with different hyperparameters, we do not believe that using a fixed number of epochs affected our results systematically. Since the number of epochs was dependent on our compute budget, we do not think that this deviation compromises the severity of our preregistered analysis.

3. **Type**: Analysis
   Reason: Plan not possible
   Timing: After data access
   Original wording: We will train LINE models directly on the Dutch population networks from 2020, 2021, 2022. We will compute first and second order embeddings with dimensions 32, 64, 128. [. . .] We will train each model until the training loss increases and at most 50 epochs.
   Deviation: Given our computational resources (i.e., the memory of available GPUs), we were not able to train LINE models with the preregistered dimensions. Instead, we trained models with 8, 16, and 24 dimensions in the first and second order embeddings, respectively. Our available computing time only allowed us to train the models for 5 epochs.
   Reader impact: The deviations were necessary due to computational constraints and thus improve the severity of our preregistered analysis by making it possible in the first place.

4. **Type**: Analysis
Reason: New knowledge
Timing: After data access
Original wording: We will train LINE models directly on the Dutch population networks from 2020, 2021, 2022. [...] We will use a learning rate of 0.1 and normal initialization for the embedding vectors.
Deviation: We also decreased the learning rate of the LINE models from 0.1 to 0.025 to make the training more stable.
Reader impact: We argue that this deviation improves the severity of our preregistered analysis because it likely led to LINE embeddings that captured the structure of the population network better.

5. **Type**: Analysis
Reason: New knowledge
Timing: After data access
Original wording: We will use the following machine learning models (estimators) for prediction: [...] Dummy regression/classifier: Uses the "prior" strategy for classification - predicts most frequent class label in the training data but returns class probabilities equal to the empirical distribution of labels in the training data.
Deviation: We omitted the dummy estimator since it would always result in predictions on "chance-level", i.e., 0.5 for AUC scores, and 0 for R-squared scores.
Reader impact: Since this deviation omitted redundant information from the analysis, we do not think that it affects its severity or inference validity.

6. **Type**: Analysis
Reason: New knowledge
Timing: After data access
Original wording: For categorical outcomes, we compute outcome class probabilities using one-vs-rest classification.
Deviation: For logistic regression, we computed outcome class probabilities using multinomial classification.
Reader impact: The deviation lead to faster training of the logistic regression models but otherwise had little impact on the severity of validity of our analysis.

7. **Type**: Analysis
Reason: Typo/error
Timing: After data access
Original wording: We will estimate models with three sets of predictors for each outcome and each estimator: The first set only includes node representations. The second set includes all covariates (age, sex, highest education, parents born abroad, purchasing power, change in purchasing power, income percentile, Big 5, happiness, mood, life satisfaction, self esteem, closeness, optimism, interpersonal trust, trust in Government [only for voting behavior outcomes]). The third set includes node representations and all covariates.
Deviation: We accidentally included whether a person did or did not vote in the covariate feature set for trust in the government.
Reader impact: Because this deviation stemmed from a programming error, we do not

think that it decreases the severity of our analysis. In the exploratory analysis, we show that whether a person voted was not an important feature for predicting trust in the government, thus, we do not think that this deviation compromises inference validity.

8. **Type**: Analysis
Reason: Plan not possible
Timing: After data access
Original wording: To evaluate whether there are differences between the scores of the models, we will estimate a Bayesian multilevel regression model with the following equations:

$$
\begin{aligned}
y_{im} =& \alpha_m + \beta_{1m}\text{estimator} + I_{\text{is\_dummy}} \times (\beta_{2m}\text{features} + \beta_{3m}\text{transformed} + \\
& \beta_4\text{year} + I_{\text{is\_deepwalk}} \times (\beta_5\text{walk\_len} + \beta_6\text{num\_walks} + \\
& \beta_7\text{walk\_len} \times \text{num\_walks} + \beta_8\text{win\_size}) + \beta_{9m}\text{emb\_dim}) + \epsilon_{y_{im}} \\
\alpha_m =& \gamma_0 + \gamma_1 u_m + \epsilon_{\alpha_m} \\
\beta_{1m} =& \gamma_2 + \gamma_3 u_m + \epsilon_{\beta_{1m}} \\
\beta_{2m} =& \gamma_4 + \gamma_5 u_m + \epsilon_{\beta_{2m}} \\
\beta_{3m} =& \gamma_6 + \gamma_7 u_m + \epsilon_{\beta_{3m}} \\
\beta_{9m} =& \gamma_8 + \gamma_9 u_m + \epsilon_{\beta_{9m}},
\end{aligned}
$$

where y_{im} is the model prediction for model i and outcome variable m. The model has a random intercept and four random slope effects for each outcome.
Deviation: The preregistered model showed poor convergence diagnostics (high R-hat values and low effective sample size for posterior inference). Therefore, we simplified the model to a univariate regression model with only fixed effects. Instead of the indicator variables, we restricted fixed effects of non-existent combinations of independent variables to zero and omit them in the output. The fixed effects are the same as in the preregistered analysis except that we added an interaction term between estimator (prediction algorithm) and features (feature set). We also added estimator and features as regressors for the dispersion parameter phi of the Beta distribution because this led to an increased posterior predictive fit.
Reader impact: This deviation impacts the severity of our preregistered analysis in two ways. Assessing diagnostic criteria and choosing a more appropriate regression model increases severity. However, choosing a model different from our preregistration also opens up many researcher degrees of freedom. By choosing a simpler model, we attempt to limit our degrees of freedom and maintain test severity.

9. **Type**: Analysis
Reason: Plan not possible
Timing: After data access
Original wording: Because the outcome variables lie between 0 and 1, we use a generalized linear model with a beta family and a logit link function.
Deviation: For the outcome trust in the government, some prediction models had R-squared scores below zero. We set these scores to zero and estimated the regression model with a zero-inflated beta family.

Reader impact: Without this deviation, we could have not estimated the regression model for prediction results for trust in the government, thus, it strengthens the severity of our analysis. While we could have chosen a different strategy for dealing with negative scores, we think that this one maintains inference validity better than, for example, excluding negative scores.

10. **Type**: Analysis
Reason: Plan not possible
Timing: After data access
Original wording: Using the representations with the highest predictive ability, we calculate a utility score for each edge and each dimension in the network (see Piaggesi et al., 2023). We then apply a threshold and select edges with a positive score which yields the subgraphs associated with each representation dimension.

We will obtain the predictive ability of the representation dimensions from the regression weights/variable importance scores of the machine learning models. For the substructure analysis, we will use the predictive ability scores of the best performing machine learning model.

From the subgraphs that are associated with highly predictive dimensions, we will create sub-subgraphs based on the levels of our outcome variables (e.g., party voted for, level of trust in government) which we will explore qualitatively and quantitatively (looking at the association of those substructures with node attributes). We will also calculate summary statistics of these subgraphs to quantify their structure and compare them to subgraphs from non-predictive dimensions.
Deviation: We selected the best performing prediction model with DINE-transformed embeddings for exploratory analysis.

We only selected the most predictive embedding dimension for substructure analysis. This dimension was determined by SHAP feature importance scores.

We did not apply the threshold and instead performed analyses on the continuous edge utility scores.

Because of privacy restrictions, we only report quantitative analyses of edge utility aggregated for different relation types and node attributes.
Reader impact: The results of this analysis are reported as exploratory. Thus, these results should not be seen as severe tests of any hypothesis.
